# Low serum insulin-like growth factor-1 in patients with erectile dysfunction

**DOI:** 10.1186/s12610-015-0028-x

**Published:** 2016-01-28

**Authors:** Alper Otunctemur, Emin Ozbek, Suleyman Sahin, Levent Ozcan, Murat Dursun, Emre Can Polat, Mustafa Cekmen, Ozgur Doga Ozsoy, Mustafa Erkoc, Eyup Danis, Muammer Bozkurt

**Affiliations:** Department of Urology, Okmeydani Training and Research Hospital, Istanbul, Turkey; Department of Urology, Derince Training and Research Hospital, Kocaeli, Turkey; Department of Urology, Istanbul Medipol University, School of Medicine, Istanbul, Turkey; Department of Biochemistry, Kocaeli University, School of Medicine, Kocaeli, Turkey

**Keywords:** Endothelial dysfunction, Erectile dysfunction, Insulin-like growth factor-1, International Index of Erectile Function Score-5, Dysfonction endothéliale, Dysfonction érectile, Insulin-like Growth Factor-1, IIEF-5

## Abstract

**Objective:**

Endothelial dysfunction and microvascular damage play a crurical role in the pathogenesis of erectile dysfunction (ED). Insulin-like growth factor-1 (IGF-1) is one of the growth factors that have a wide range of biologic effects. IGF-1 is an important mediator of cell growth, differentiation and transformation in various tissues. The purpose of the current study was to determine the association between IGF-1 levels and ED.

**Materials and methods:**

All men were evaluated for ED and divided into two groups: 80 patients suffering from ED for > 1 year and 80 subjects without ED were enrolled as a control group in this study. Diagnosis of ED was based on the International Index of Erectile Function Score-5. IGF-1 levels were measured in serum by an automated chemiluminescence immunoassay. The relationship between IGF-1 levels and ED scores in patients was statistically evaluated.

**Results:**

The mean age of patients in ED group was 60.4 ± 11.3 years and 55.4 ± 9.6 in control group. The plasma IGF-1 levels were significantly lower in ED than in control group (96.5 ± 38.3 and 132.5 ± 53.3 ng/ mL, respectively, *P* < 0.001). The IGF-1 levels were positively correlated with ED score (r = 0.623, *P* < 0.01).

**Conclusion:**

In this study serum IGF-1 levels were found to be associated with endothelial dysfunction that predicts ED. Serum IGF-1 level appears to be a specific predictor of ED, and it might be used in early prediction of ED in male population.

## Résumé

**Objectifs:**

La dysfonction endothéliale et les altérations vasculaires jouent un rôle crucial dans la pathogenèse de la dysfonction érectile (DE). L’Insulin-like Growth Factor-1 (IGF-1) est l’un des facteurs de croissance qui ont un vaste champ d’effets biologiques. IGF-1 est un médiateur important de la croissance, différentiation et transformation cellulaires dans différents tissus. Le but de la présente étude était de rechercher une association entre les taux d’IGF-1 et la dysfonction érectile.

**Matériel et Méthodes:**

La dysfonction érectile a été évaluée chez tous les hommes, constitués en 2 groupes : 80 patients souffrant de DE depuis plus d’un an, et 80 sujets sans DE recrutés comme groupe témoin dans l’étude. Le diagnostic de DE était basé sur le score 5 de l’Index International de la Fonction Erectile. Les taux d’IGF-1 ont été mesurés par immunodosage automatisé par chemiluminescence. La relation entre les taux d’IGF-1 et le score de la DE a été statistiquement évaluée.

**Résultats:**

L’âge moyen des patients du groupe DE était de 60.4±11.3 ans et de 55.4±9.6 dans le groupe témoin. Les taux plasmatiques d’IGF-1 étaient significativement plus bas dans le groupe DE que chez les témoins (respectivement 96.5±38.3 et 132.5±53.3 ng/mL, P < 0.001). Les taux d’IGF-1 étaient positivement corrélés au score de la DE (r = 0.623, P < 0.01).

**Conclusions:**

Nos résultats indiquent que les taux sériques d’IGF-1 sont associés à une dysfonction endothéliale qui prédit la DE. L’IGF-1 sérique apparaît être un prédicteur spécifique de la DE, et il pourrait être utilisé pour une prédiction précoce de la DE dans la population masculine.

## Background

Erectile dysfunction (ED) is defined as the recurrent or consistent inability to obtain and maintain a penile erection sufficient for satisfactory sexual performance, it represents a common public health problem that significantly impairs the quality of life and psychological well-being of the patient and his partner [1]. The incidence and the severity of ED increase with age, reaching 20–40 % in men 60–69 yr of age and 50–100 % in men in their 70s and 80s, depending on the differing definitions of ED in various studies [2]. Recent data demonstrate that ED is associated with impaired endothelial-dependent flow-mediated dilatation (FMD), suggesting that ED is associated with endothelial dysfunction [3]. The etiology of ED is usually multifactorial, with vascular, aging, hormonal, neurologic, and psychological factors playing roles [4]. The common risk factors, diabetes mellitus, hypertension, obesity, dyslipidemia, smoking and sedentary lifestyle, for endothelial dysfunction and atherosclerosis have been frequently found in patients with ED [5].

Insulin-like growth factor (IGF)-1, a peptide hormone that is structurally related to insulin and synthesized by almost all tissues, is an important mediator of cell growth, differentiation and transformation in various tissues [6]. IGF-1 is a potent mitogen and antiapoptotic factor for cell types and exerts all of its known physiologic effects by binding to the IGF receptor (IGF-1R) [7]. IGF binding activates IGF-1R, which in turn phosphorylates phosphatidylinositol 3-kinase (PI-3K) and Ras ⁄ Raf ⁄ mitogen-activated protein kinase (MAPK). Ras ⁄ Raf ⁄ MAPK and PI-3K play important roles in IGF1R- induced cellular proliferation and the inhibition of apoptosis [8]. IGF-1 plays a crucial role in the regeneration of nitric oxide synthase (NOS) containing nerve fibers in the dorsal and intracavernosal nerves and administration of IGF-1 can facilitate the regeneration of NOS containing nerve fibers in penile tissue and enhance the recovery of erectile function after bilateral cavernous nerve cryoablation [9, 10]. In diabetic rats with ED, downregulation of IGF-1 protein expression was found in penile corpus cavernosum [11].

Because of the wide range of their biologic effects and therapeutic potential, involvement in a number of disease processes, the IGF-1 has become the focus of research by an increasing number of investigators. The aim of this study was to determine whether any relationship exists between ED and the level of IGF-1.

## Methods

We performed a prospective study of participants who visited Okmeydani Training and Research Hospital from March 2011 to January 2013. Before the begining of the study all patients were evaluated for ED and divided into two groups: 80 patients suffering from ED for >1 year and no history of penile surgery or pelvic trauma were enrolled as a study group and 80 subjects without ED were enrolled as a control group. Controls are healthy persons working in the hospital who volunteered for the study. Local ethics committee approval from Okmeydani Training and Research Hospital had been obtained before the commence of the study.

All men had a complete detailed and careful history taken, with special attention to the sexual history, including details to differentiate between psychogenic and organic ED; a complete physical examination, including genital and neurological examination; blood glucose assay, urine analysis, complete blood assessment, and kidney and liver function; hormonal assays of testosterone, prolactin, and thyroxin; combined intracavernosal injection and stimulation with a standard dose of 1-mL papaverine HCl (30 mg). Erectile function was assessed using the abridged five-item version of the International Index of Erectile Function questionnaire (IIEF-5), a validated, self administered questionnaire. Possible scores for the IIEF-5 range from 5 to 25; scores of 22–25 indicate normal erectile function, while scores of 21 or below indicate ED [12]. According to the IIEF-5 score, ED can be classified as severe (5–7), moderate (8–11), mild-to-moderate (12–16), or mild (17–21).

Exclusion criteria included: congestive heart failure (ejection fraction <50 %), pulmonary hypertension, stroke, known peripheral atherosclerotic disease, surgical coronary intervention, percutaneous coronary angioplasty and/or stenting, stable and unstable angina pectoris, impaired renal function (creatinine > 1.4 mg/dL), unstable endocrine or metabolic diseases (e.g., hypoprolactinemia and hyperprolactinemia, hypoandrogenic and hyperandrogenic states, hypothyroidism and hyperthyroidism), and patients with concomitant inflammatory diseases such as infections and autoimmune disorders, patients with Peyronie disease acute/chronic hepatic or hepatobiliary disease, and malignancy. Patients who underwent radical prostatectomy- pelvic surgery, patients with abnormal nutritional status or eating disorders and taking beta-blocker, spironolactone, corticosteroids, antioxidant vitamins, and alcohol were also excluded from the study.

Blood samples of all patients were taken from an antecubital vein following an overnight fasting state. Serum IGF-1 levels were measured by an Automated chemiluminescence immunoassay (Immunodiagnostic Systems Limited, Boldon, Tyne & Wear, UK). Assays for serum triglyceride (TG), total cholesterol (Total-C), low-density lipoprotein cholesterol (LDL-C), and high-density lipoprotein cholesterol (HDL-C) levels were performed in the hospital’s chemistry laboratory.

### Statistical analysis

Statistical analyses were performed by the Statistical Package for Social Sciences, version 15.0, software (SPSS Inc., Chicago, IL, USA). The quantitative demographic values were evaluated by student’s t or Mann Whitney U test whether the parameters were suitable for normal distribution or not. If the parameters are qualitative chi-square test was used. ANOVA test was conducted to evaluate the difference between subgroups of the patients stratified for ED. Spearman correlation test was performed to analyse the association between ED and IGF-1 level. All tests were performed using a 2-tailed analysis, and a *P* value of < 0.05 was considered statistically significant.

## Results

**Fig. 1:**
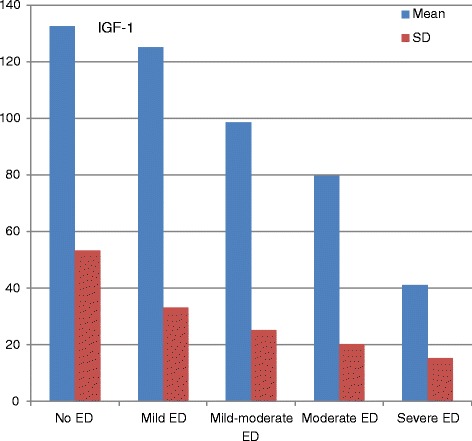
Distributions of IGF-1 levels according to the degree of erectile dysfunction (ED). Values are means (±SD) of IGF-1: 132.5 ± 53.3 [no; *n* = 80], 125.1 ± 33.2 [mild; *n* = 29], 98.6 ± 25.1 [mild-moderate; *n* = 24], 79.6 ± 20.2 [moderate; *n* = 16] and 41.1 ± 15.3 [severe; *n* = 11], respectively, *p* < 0.001

A total of 160 men between the ages of 45 and 70 years were analysed and divided into the two groups: 80 patients with ED and 80 subjects without ED (control). The baseline characteristic properties of study men were demonstrated in Table 1. The mean age was 60.4 ± 11.3 years in patients with ED group and 55.4 ± 9.63 years in control group. There were no significant differences between two groups with respect to age, prevalence of diabetes mellitus, hypertension, smoking and levels of fasting blood glucose, serum creatinine, Total-C, LDL-C, HDL-C, TG (*P* > 0.05 for all). The mean value of body mass index (BMI) in patients with ED was 29.6 ± 3.1 and in control group was 25.9 ± 2.3. There was statistically difference for BMI between groups. We compared the IGF-1 levels between groups. Our results showed that the patients with ED have lower IGF-1 levels than control group. Mean IGF-1 level was 96.5 ± 38.3 in patients with ED and 132.5 ± 53.3 in control goup, respectively. There was statistically difference for IGF-1 levels between the patients with ED and control group (*p* < 0.001) (Table 1). The IGF-1 levels were positively correlated with ED score (*r* = 0.623, *p* < 0.01). The IGF-1 levels were significantly decreased with the grade of ED as mild-to-severe (*p* < 0.001) (Fig. 1).

**Table 1:** Baseline characteristic properties of study patients and controls

	ED group (*n* = 80)	Control group (*n* = 80)	*P* value
Age (years)	60.4 ± 11.3	55.4 ± 9.6	0.07
Diabetes mellitus (%)	43	36	0.15
Hypertension (%)	37	30	0.20
Smoke (%)	63	57	0.35
Fasting blood glucose (mg/dl)	114.4 ± 35.4	106.7 ± 28.8	0.32
Creatinin (mg/dl)	0.80 ± 0.3	0.77 ± 0.5	0.10
Trigliseride (mg/dl)	191.5 ± 77.8	185.7 ± 71.8	0.40
Total cholesterole (mg/dl)	214.3 ± 23.5	209.6 ± 21.6	0.33
LDL-C (mg/dl)	132.2 ± 18.5	124.3 ± 18.7	0.20
HDL-C (mg/dl)	43.4 ± 10.2	46.9 ± 11.7	0.37
IGF-1 level (ng/ml)	96.5 ± 38.3	132.54 ± 53.3	<0.001*
BMI	29.6 ± 3.1	25.9 ± 2.3	0.001*
ED score	12 ± 1.2	23 ± 0.8	<0.001*

Values are means (±SD) or percentages

*BMI* Body mass index, *ED* Erectile dysfunction, *HDL-C* HIgh-density lipoprotein cholesterol, *LDL-C* Low-density lipoprotein cholesterol, *IGF-1* Insulin like growth factor-1

*P < 0.05 was accepted as statistically significant

## Discussion

The purpose of this investigation was to examine the association between IGF-1 levels and ED in men. The results of this study showed that the patients with ED had significantly lower IGF-1 levels than control group. Furthermore, our study revealed that there was a positive correlation between IGF-1 levels and ED score. To our knowledge, this is the first study to show that the serum levels of IGF-1 represent a marker of ED patients. This study suggested that IGF-1 might contribute to the pathophysiology of ED in patients. In a previous study, IGF-1 levels were lower in diabetic men [13]. In another study done by Castela A et al. they found that significant reduction IGF-1 protein expressions in human diabetic samples [14]. Also, we found that IGF-1 levels were lower in men with ED; however in our series, DM prevalence is similar between men with and without ED. In this context, IGF-1 levels were lower in men with ED independently from DM.

ED is caused by two main etiologies: organic and psychogenic. Organic causes of ED comprise up to 80 % of cases and vascular disease is the most common pathophysiology of ED [15]. It was generally accepted that ED has been associated with advanced atherosclerotic disease [16]. IGF-1 suppresses atherosclerosis and anti-atherosclerotic effect was associated with a reduction in vascular and systemic oxidative stress, an increase in circulating nitric oxide (NO) bioavailability and vascular endothelial nitric oxide synthase (eNOS) expression, the main NO-producing enzyme in the vascular wall [17]. Also IGF-1 reduced total cell apoptosis in the atherosclerotic plaque and increased features of atherosclerotic plaque stability [18]. Contrary to its anti-oxidant effect, antiapoptotic and plaque stability effect of IGF-1 was NO-independent [12]. But, several other studies have also reported that endothelial dysfunction in the penile circulation accompanies ED in association with various cardiovascular risk factors without advanced atherosclerotic disease [19, 20].

IGF-I shows high affinities for insulin-like growth factor binding proteins (IGFBPs). IGFBP-3 carries most of the circulating IGF-I. IGFBP-3 regulates the availability of IGF-I by restricting the extravascular transit of IGF-I to the target cells [21]. It is confirmed that increases in IGFBP-3 expression due to diabetes would attenuate the cellular response to IGF-1 through the high affinity binding of IGF-1 to IGFBP-3. This would result in decreased availability of IGF-1 for the receptor and may result in ED [22]. Furthermore, a significant decrease in the amount of IGF-1 gene expression in penile cavernosum of the diabetic rats with ED was confirmed [11]. IGFBP-3 was detected excess in the penile endothelium and in the smooth muscle of the corpus cavernosum of the aged rats and diabetic rats [23]. Both the increased IGFBP-3 and decreased IGF-1 contributed to decrease the availability of IGF-1 in erectile tissue and resulting ED.

The corporal cavernosal smooth muscle plays a major role in modulating penile blood flow during erection and detumescence [24]. It has been confirmed that erectile dysfunction is a result of trabecular structural changes, including cavernosal smooth muscle cell atrophy and excess connective tissue accumulation [25]. In a study the amount of smooth muscle in the corpus cavernosum was used to evaluate the erectile function. The results show that IGF- 1 was effective for maintaining the amount of smooth muscle. The percentage of smooth muscle in the corpus cavernosum seemed to increase with IGF-1 treatment [26]. It is also suggested that the decrease in IGF-1 is associated with decreased NOS activity and cavernosal cyclic guanosine monophosphate (cGMP) levels as well as the decreased smooth muscle integrity, thus impairing cavernosal smooth muscle relaxation with a resultant decrease in erectile function [23].

Endothelial dysfunction is characterized by an imbalance between the endothelium-dependent vasodilator and vasoconstrictor activity, and is associated with a proinflammatory, proliferative, and procoagulatory environment [27]. Increasing evidence suggests that endothelial dysfunction is a systemic disorder and implicated in the pathogenesis of ED, affecting both conduit arteries and microvessels in various vascular beds [28]. The key role that regulates endothelial function is endothelial-derived NO [27]. Endothelial NO is important in producing the arterial and venous dilation necessary to attain and sustain an erection. Abnormalities of this vasodilator system are present in atherosclerosis and play an important role in the pathophysiology of ED. The penile vascular bed is dependent on NO for vasodilatation of the arteries to produce rapid blood inflow and vasodilatation of the trabecular smooth muscle of the lacunar space to prevent venous outflow [29]. Dysfunctional endothelial cells lining the penile arterial system and the corpus cavernosum produce less NO. Impaired NO activity appears to play a crucial role in both endothelial dysfunction and ED [30]. IGF-1 has been shown to regulate circulating NO bioavailability and vascular endothelial NOS expression in endothelial cells [31]. IGF-I induces NO-dependent dilation in the vascular beds. Decreased IGF-1 levels with aging and diabetes reduce penile NOS expression and reduced availability of NO are among the causal factors implicated in ED [32]. IGF-I has roles in cell proliferation, migration, and suppression of apoptosis to preserve cellular integrity [33]. Endothelial progenitor cells (EPCs) promote endothelial repair, thereby preventing endothelial dysfunction [34]. eNOS, which is crucial for maintenance of endothelial function, is also involved in mobilization of EPCs from bone marrow. It has been shown in human subjects that an increase in circulating IGF-1 or IGF-1 administration raised circulating EPC numbers and this was associated with increased NO bioavailability [35].

Part of the allure of IGF-1 as a therapeutic agent is its wide range of biologic effects and its actions on many different tissues. Therapeutic effects of IGF-1 on ED have been studied in animal experiments. Pu et al. [26] showed that intracavernosal injection of IGF-1 ameliorated erectile responses in aged rats. The penile responses were examined as significant increases in intracavernous pressure. The erectile responses restored by IGF-1. IGF-1 treatment increased not only the percentage of smooth muscle, but also increased the expression of eNOS, NOS activity and cGMP concentration in the penile tissues. They suggested that the major mechanism underlying the therapeutic effects of IGF-1 involves increasing the integrity of smooth muscle and modulating the NO-cGMP signaling pathways.

The role of oxidative stress has been highlighted in in vitro studies showing that increased production of reactive oxygen species is associated with decreased normal erectile response primarily because of inactivation of NO [36, 37]. Recent studies suggested that endothelium is a main source of reactive oxygen species (ROS) [38]. Low IGF-1 serum concentrations were associated with high oxidative stress in the vasculature, potentially leading to endothelial dysfunction [39]. IGF-1 reduced superoxides and enhanced expression of antioxidant enzymes such as Mn-superoxide dismutase (SOD), Cu/Zn-SOD and glutathione peroxidase in animal experimental. The IGF-1-induced antioxidant effect was NO-dependent (at least in part) since the ability of IGF-1 to reduce oxidative stress was significantly blunted by L-arginine methyl ester hydrochloride (L-NAME) [39, 40].

The lack of insulin levels and insulin resistance index by homeostasis model assessment (HOMA-IR) determinations is a limitation of this study.

## Conclusion

This study shows a relationship between decreased serum IGF-1 level and the presence and severity of ED, which may be postulated as evidence of endothelial dysfunction in ED. Serum IGF-1 level might be used in early prediction of ED in future and it may be used in early prediction of ED in male population. However, further clinical studies are needed to clarify the pathophysiological role of serum IGF-1 in ED patients.

## Abbreviations

C: cholesterol
cGMP: cyclic guanosine monophosphate
ED: erectile dysfunction
eNOS: endothelial nitric oxide synthase
EPC: endothelial progenitor cell
FMD: flow mediated dilatation
HDL: high density lipoprotein cholesterol
HOMA-IR: insulin resistance index by homeostasis model assessment
IGF-1: insulin like growth factor-1
IGF-1R: insulin like growth factor receptor-1
IGFBP: insulin like growth factor binding protein
LDL: low density lipoprotein cholesterol
MAPK: mitogen activated protein kinase
NO: nitric oxide
NOS: nitric oxide synthase
PI-3K: phosphatidylinositol 3- kinase
ROS: reactive oxygen species
SOD: superoxide dismutase
TG: trigilyceride
